# The Effect of Various Waste Materials’ Contents on the Attenuation Level of Anti-Radiation Shielding Concrete

**DOI:** 10.3390/ma6104836

**Published:** 2013-10-23

**Authors:** Ali Basheer Azeez, Kahtan S. Mohammed, Mohd Mustafa Al Bakri Abdullah, Kamarudin Hussin, Andrei Victor Sandu, Rafiza Abdul Razak

**Affiliations:** 1 Centre of Excellence Geopolymer & Green Technology (CEGeoGTech), School of Material Engineering, Universiti Malaysia Perlis (UniMAP), P.O. Box 77, D/A PejabatPosBesar, Kangar, Perlis 01000, Malaysia; E-Mails: kahtan@unimap.edu.my (K.S.M.); vc@unimap.edu.my (K.H.); rafizarazak@unimap.edu.my (R.A.R.); 2 Faculty of Materials Science and Engineering, Gheorghe Asachi Technical University of Iasi, Blvd. D. Mangeron 71, Iasi 700050, Romania; E-Mail: sav@tuiasi.ro

**Keywords:** attenuation coefficient, radioactive, wastes Iron particulates, concrete, NaI (Tl), steel slag, steel ball

## Abstract

Samples of concrete contain various waste materials, such as iron particulates, steel balls of used ball bearings and slags from steel industry were assessed for their anti-radiation attenuation coefficient properties. The attenuation measurements were performed using gamma spectrometer of NaI (Tl) detector. The utilized radiation sources comprised ^137^Cs and ^60^Co radioactive elements with photon energies of 0.662 MeV for ^137^Cs and two energy levels of 1.17 and 1.33 MeV for the ^60^Co. Likewise the mean free paths for the tested samples were obtained. The aim of this work is to investigate the effect of the waste loading rates and the particulate dispersive manner within the concrete matrix on the attenuation coefficients. The maximum linear attenuation coefficient (μ) was attained for concrete incorporates iron filling wastes of 30 wt %. They were of 1.12 ± 1.31×10^−3^ for ^137^Cs and 0.92 ± 1.57 × 10^−3^ for ^60^Co. Substantial improvement in attenuation performance by 20%–25% was achieved for concrete samples incorporate iron fillings as opposed to that of steel ball samples at different (5%–30%) loading rates. The steel balls and the steel slags gave much inferior values. The microstructure, concrete-metal composite density, the homogeneity and particulate dispersion were examined and evaluated using different metallographic, microscopic and measurement facilities.

## 1. Introduction

Day by day radiation protection becomes more and more important topic to be investigated in nuclear science. Shielding from gamma rays is more difficult than others because gamma photons have no mass and charge and hold high-energy, they can readily penetrate into the matter [1]. One of the most widely used materials in reactor shielding is concrete; it is basically a mixture of Portland cement, sand, coarse aggregates and water. It is cheap, easy to prepare in different compositions, and easy to form and to use in construction works. Radiation shielding concrete can be used to attenuate both neutron and gamma rays. The photon interaction with the matter depends on the incoming photon energy and the density of the shielding material [2]. The concrete shielding properties may vary depending on the material components of the concrete. Aggregates are the largest constituent (about 70%–80% of the total weight of normal concrete) [3].

Heavy concrete materials are those with the density greater than 2600 kg/m^3^. Portland cement is the main cementitious component of conventional concrete. Several papers are found working on improvement of concrete properties to suit the shielding requirements. They mostly concentrate on reducing the water contents, using other cementitious materials such as steel blast furnace slags, silica fumes and polymeric compounds rather than Portland cement [3,4]. In order to achieve higher density, some researchers try to entirely or partially replace the traditional fillers of concrete (sand, gravel, crushed rocks) with materials possess larger specific gravity such as magnetite, hematite, barite and colemanite [5,6,7]. On the other hand, very few of these papers are on the addition of metal particulates to concrete to enhance its density to be used for shielding purposes. Kan *et al*. [8] worked on the effect of various iron aggregate inclusions on strength and fracture toughness of heavy concrete. They found that addition of 40% metallic aggregate content by volume enhances strength and fracture toughness and prevent cracking of shielding walls in reactor vessels. Maslehuddin* et al.* [9] studied the effect of incorporating electric arc furnace slag aggregates (EAFSA) and steel shots on the attenuation performance and mechanical properties of conventional concrete. They reported that admix of 50 wt % of EAFSA and 50 wt % of steel shots with concrete meets the weight and radiation requirements and their usage result in substantial cost reduction.

For long time lead was used for anti radiation protection whether alone or within concrete walls. Recently the toxicity of lead steered researchers to look for alternative materials. Other materials such as steel in paraffin/poly-ethylene, hydrogen, silicon or carbon, boron and depleted uranium were proposed for anti radiation protection [10,11,12]. These materials are not easy to be processed, relatively expensive, not abundant. Some others are expected to cause cancer like depleted uranium.

In composite materials, a single number cannot represent the atomic number as in the case of an element. This number is defined as the “effective atomic number” and it is a convenient parameter for evaluation of photon interaction with the medium [13].

Jameel *et al.* [14] calculated the linear attenuation coefficient of two types of shielding materials made from Saudi white and red sands. Their study showed that white sand is better than red sand for attenuation gamma-ray.

In this work, granules of iron fillings wastes, slags and/or iron balls of used ball bearings are proposed as additives. Slags of blast furnaces from steel industry were added to the concrete mix as cementitious material. Huge quantities of by-product materials can be reused in this type of concrete. Better performance can be attained by manipulating particulate size, distribution and orientation of these metal contents.

To assess the attenuation degree, gamma spectrometer of NaI (Tl) detector was used. The utilized radiation sources comprise ^137^Cs and ^60^Co radioactive elements. The photon liner attenuation coefficients (μ) and the mass attenuation coefficients (μ/ρ) will be measured using computer code software.

The long range target of this study is to develop a cheap concrete-metal composite based on recycled materials of effective shielding properties to be used in construction of warehouses for nuclear wastes and private rooms for X-ray and radiation therapy equipment in medical fields.

## 2. Experimental

**Figure 1:**
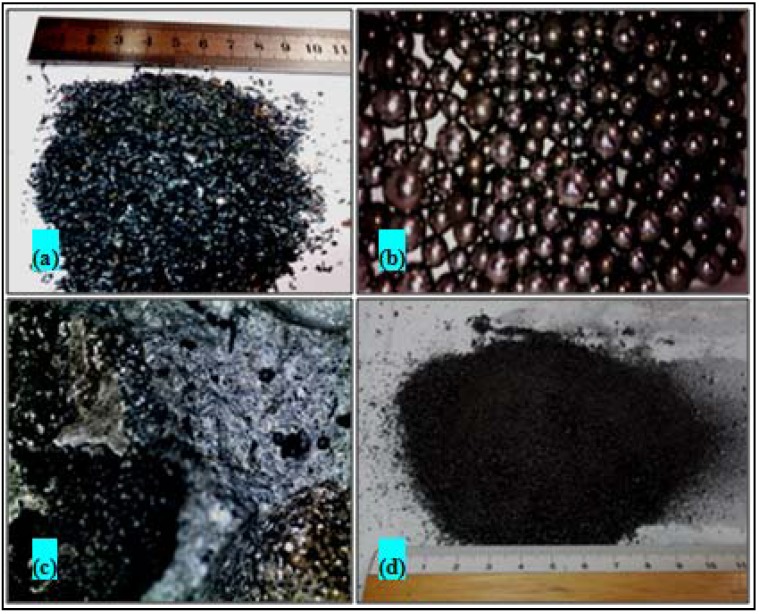
Low magnification optical graphs of the metal particulates and the steel slags used in this study: (**a**) Iron particulates from abrasives machining of steel; (**b**) Steel balls 2 to 6 mm in size; (**c**) steel slags before crushing; (**d**) crushed and screened steel slags.

Three concrete sample sets consisted of five samples each of different waste material contents incrementally increased from 5 to 30 wt %. These materials included iron particulates, steel balls of used ball bearings and slags from steel industry. The iron particulates and the steel balls were collected from different workshops in school of material building and from the industrial area in Penang; they were debris from mechanical turning, milling and abrasive machining of steel, mainly mild steel. The metal particulates passed through degreasing, cleaning and milling before usage. The steel balls were acquired from dismantling used ball bearings collected from industrial area in Penang. They were decreased and cleaned before usage. The steel blast furnace slags supplied by Ann Joo Resources Berhad Steel Company in Betaling Jaya Kuala Lumpur. The iron and the steel slags particulates were refined and sieved to about 188 μm. Figure 1 shows the features of the particulates used in this study. Before molding, the concrete-particulate admixture was mixed for 20 min which were considered enough to achieve good homogeneity. The bulk density (*ρ*) of the prepared samples was evaluated by weight and direct sample dimensions measurement.

The attenuation measurements were performed for each sample using gamma spectrometer of NaI (Tl). The measurements were performed using gamma ray spectrometer of 3ʺ × 3ʺ NaI (Tl) detector with a Multi Channel Analyzer (MCA) .The spectrometer communicates with the PC by Genie200 software.

**Figure 2:**
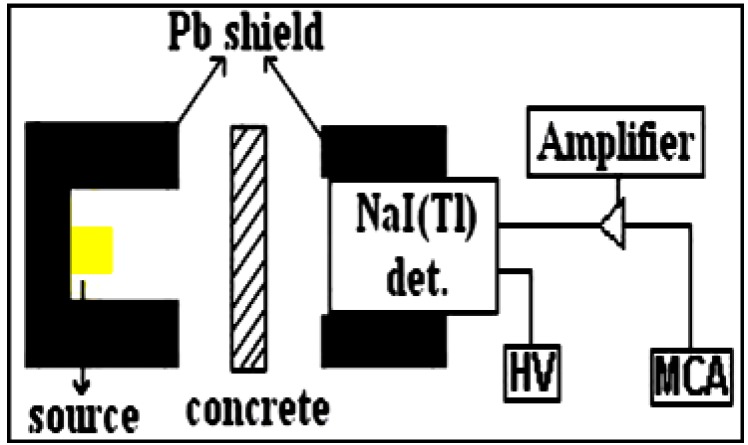
Schematic representation of the experimental setup.

The emitting photon energies of the utilized ^137^Cs and ^60^Co sources were of 0.662, 1.173 and 1.332 MeV respectively. The linear attenuation coefficients for these samples were experimentally determined using narrow collimated mono-energetic beam of gamma rays. The schematic representation of the experimental setup is shown in Figure 2.

### Calculations

**Figure 3:**
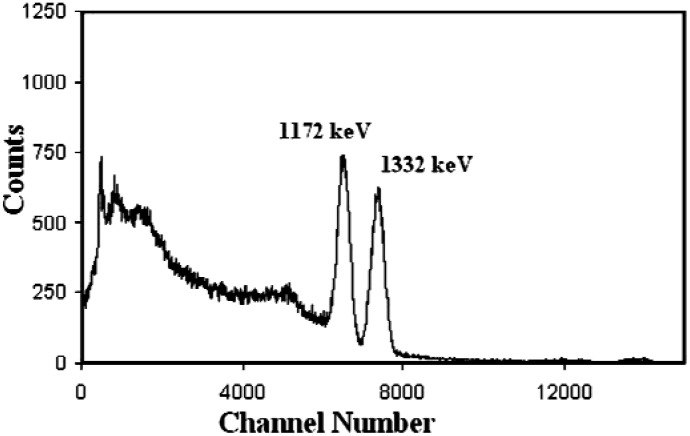
Gamma ray spectrum obtained from ^60^Co source.

Background was subtracted from the initial intensity (*I*_0_) and the Intensity (*I*) of the transmitted beam. The density (ρ) is mass and volume dependant. The linear attenuation coefficients μ was determined by measuring the transmission of gamma-rays through a target and sample of known thickness. Gamma ray spectrum for ^60^Co is shown in Figure 3, wherein the two sharp peaks related to 1.17 and 1.33 MeV gamma rays can be seen clearly. The area under the curve of the photo peak spectrum is used to evaluate the intensity *I* of the transmitted beam. Evaluation of *I*_0_ which is the area under the photo peak is obtained without inserting any sample between the detector and the source, from *I* and the incident photon *I*_0_ for a thickness *x* of the absorber, the linear attenuation coefficients μ is given by the following formula:

(1) $$\mu \text{=}\frac{1}{x}\ln\frac{I_{0}}{I}$$

Equation (1) is valid only if two conditions are satisfied. First; the photons in the incident beam are mono-energetic. Second, the beam must be narrow. For broad beam source geometry or thick shield, usually another term is introduced to Equation (1) called the buildup factor *B* [15].

The mean free path (mfp) is the average distance the photon travels between collisions with the atoms of the target material. It can be taken as the length of the path divided by the number of collisions. The mfp depends on the material characteristics and the energy of the radiated photons [16]. It is related to the attenuation by:

*mfp =* µ^−1^ (2)

## 3. Results and Discussion

As a solid waste management practices, Zainab and Enas [17] examined the feasibility of reusing iron waste of different wt % as a partial replacement of sand in concrete. They reported that as the content of the iron waste increases the fresh and dry density values of waste-iron concrete mixes tend to increase while the slump values of the iron concrete mixes decreases. Bouzarjomehri* et al.* [18] produced heavy concrete samples using barite mineral. The samples they made had densities in the range of 3.1–3.5 g/cm^3^. The measured half value layer (HVL) of their samples for ^60^Co gamma radiation source of 1.25 MeV was 3.6–4.0 cm. They reported that the performance of this concrete mix for gamma ray attenuation is substantially better than conventional concrete.

**Figure 4:**
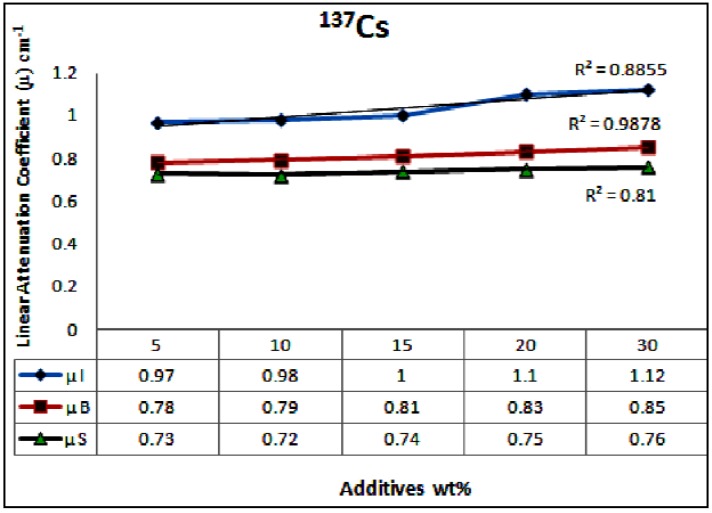
The average linear attenuation coefficients of the examined samples as a function of wt % of the waste materials, for ^137^Cs energy, µI, µB and µS are the attenuation coefficients for the samples of iron particulates, steel balls and steel slags, respectively.

**Figure 5:**
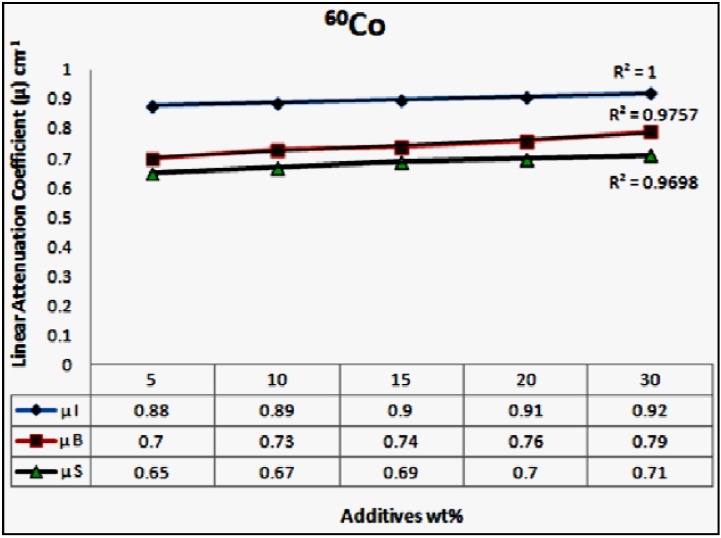
The average linear attenuation coefficients of the examined samples as a function of the wt % of the waste materials, for the ^60^Co energy source, µI, µB and µS are the attenuation coefficients for the samples of iron particulates, steel balls and steel slags, respectively.

In this investigation the linear attenuation coefficients of concrete containing different wt % of wastes materials up to 30 wt % were measured. The results are displayed in Figure 4 for ^137^Cs source and in Figure 5 for ^60^Co source, it can be seen that the linear attenuation coefficients increased with increasing the content of waste materials in the concrete samples. Samples containing iron particulates exhibited the highest values for linear attenuation coefficients as opposed to other samples.

The correlation between the linear attenuation coefficients and the wastes materials content in the concrete is used to confirm the linearity. The plots in Figure 4 and Figure 5 showed that the correlation coefficients (*R*^2^) for ^137^Cs energy source of 0.662 MeV are 0.89, 0.99 and 0.81 for iron particulates, steel balls and steel slags, respectively. The *R*^2^ values for ^60^Co energy source of 1.17 and 1.33 MeV are of 1.0, 0.98 and 0.97 for iron particulates, steel ball and steel slags, respectively.

**Figure 6:**
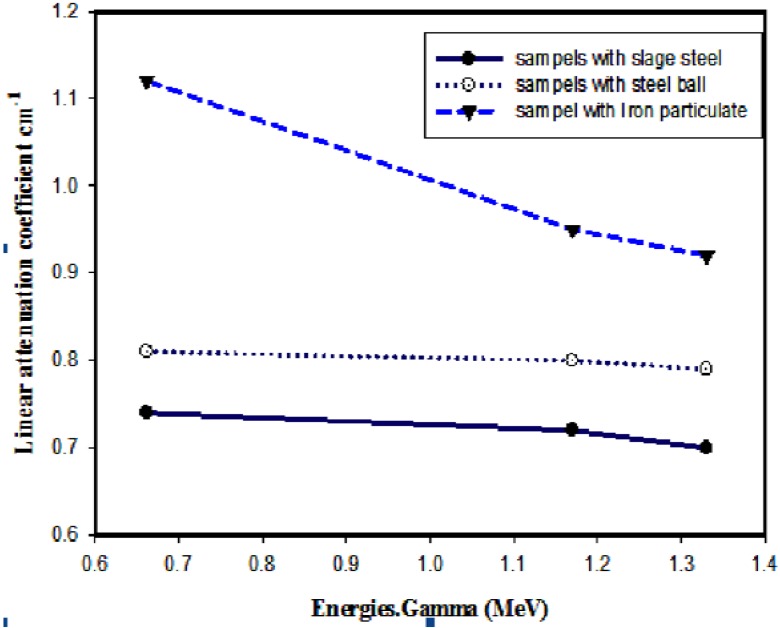
The average linear attenuation coefficients of the examined samples as function of Gamma-ray photon energy.

It is obvious that the content of the high dense waste material component within the concrete mix is effective in suppressing the transmission of radiation. Comparing the *μ* values of concrete samples for the ^137^Cs and ^60^Co sources in Figure 5 and Figure 6 reveals that substantial improvement in attenuation performance by 20%–25% was achieved for concrete samples incorporate iron fillings as opposed to that of steel ball samples at different (5%–30%) loading rates.

The variation of the linear attenuation coefficients against photon energy for ^137^Cs gamma ray source is exposed in Figure 6. The group containing iron particulates gave the best results whereas the attenuation coefficients decrease as the photon energy is increased.

**Figure 7:**
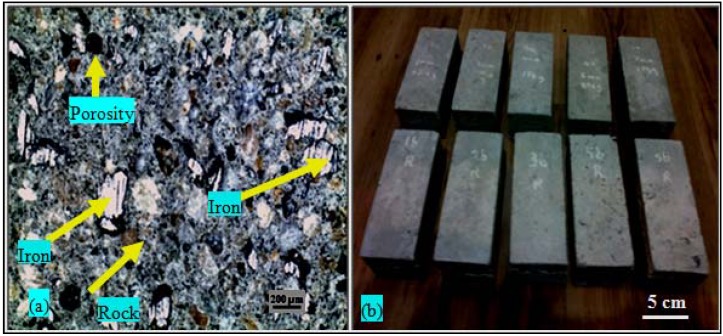
(**a**) A low magnification cross sectional optical macrograph of concrete mold of 30 wt % iron particulates exhibits the distribution of the iron particulates within the concrete matrix; (**b**) optical graphs of the tested samples of this study.

Figure 7 depicts the features of the tested samples. Figure 7a shows the macrostructure of the concrete-iron waste particulates. The iron particulates are evenly arranged and distributed within the concrete mix. Figure 7b shows different samples of concrete with different steel slags, iron waste particulates and steel balls contents.

**Figure 8:**
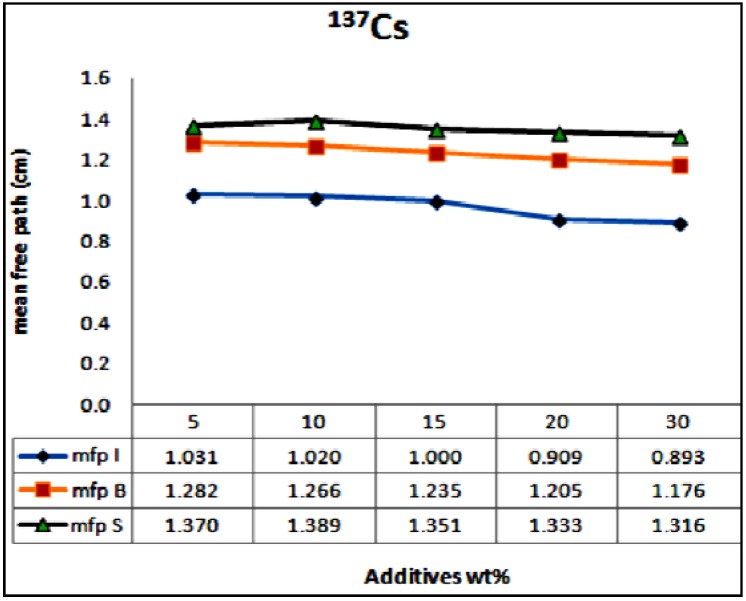
Variation of mfp as a function of the additives wt % for the^137^Cs source, µI, µB and µS standing for the attenuation coefficients for the tested samples of iron particulates, steel balls and steel slags, respectively.

**Figure 9:**
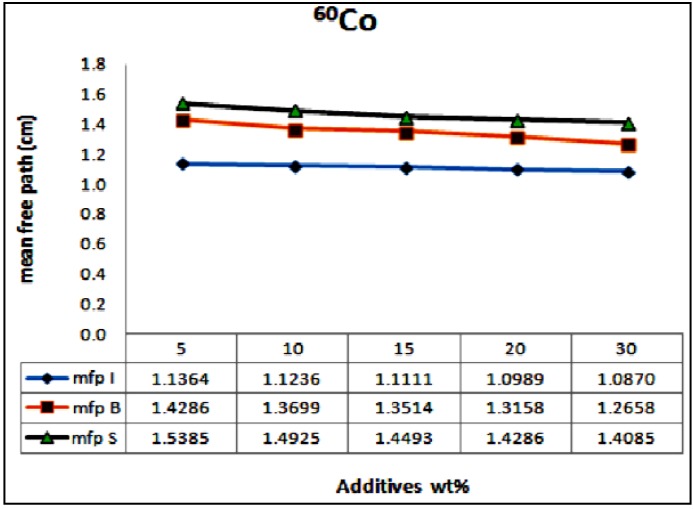
Variation of mfp as a function of the additives wt % for the ^60^Co source, µI, µB and µS standing for the attenuation coefficients for the samples of iron particulates, steel balls and steel slags, respectively.

The mean free path (mfp), is the reciprocal of the measured linear attenuation coefficients. It is plotted* versus* different wt % of the waste materials at different photon energies of 0.662, 1.17 and 1.33 MeV. The results are displayed in Figure 8 and Figure 9, for ^137^Cs and ^60^Co, respectively. These figures indicate that concrete sample of lower wt % of iron waste material suppresses the incident radiation and attenuates it within longer distance,* i.e.*, higher mean free path (mfp) and consequently higher HVL values as opposed to sample of higher wt % of iron waste materials which takes relatively shorter distance to attenuate the same radiation. It is common that increasing the relatively heavy metal contents within concrete improves its bulk density. The density of the anti radiation shielding material is an important factor in attenuation. Hence reduction of porosity levels and incorporating particulates of high specific gravity enhance the attenuation performance of concrete. The authors elsewhere [19] studied the effect of aggregate size on the attenuation performance of concrete utilizing different radiation sources. They reported that concrete samples of mixed aggregates gave the higher linear attenuation coefficients as opposed to the other tested samples. They attributed that to the high close packing level of the mixed aggregates which consequently lead to the reduction in porosity level and improvement in the bulk density of the concrete mix.

However, increasing density alone is not enough to develop an efficient shielder. The heavy particulate size distribution and orientation have enormous effect on attenuation. Attenuation process mainly relies on the probability of collision of the incident rays with the heavy particulates within the matrix and this usually expressed by the mfp value. Evenly dispersed coherent particulates tremendously enhance attenuation. This argument is supported by the plots in Figure 8 and Figure 9. Both iron balls and iron particulates have the same density (7.85 g/cm^3^), if their wt % within the concrete sample is kept same, the bulk density of their concrete mix samples should be similar and consequently from density perspective their attenuation performance should be similar as well. But this is not true, the samples of concrete-steel balls mix consistently showed higher mfp,* i.e.*, lower attenuation coefficient values at similar loading rates irrespective of the radiation source as compared to that of iron waste particulates. This attributed to the higher probability of collision or interaction between the incident rays and the relatively small well dispersed iron particulates within the sample as opposed to that of steel balls. In other words, besides density, dispersion and particulates/matrix areal ratio are other crucial parameters have to be considered in designing an efficient anti radiation composite material.

**Figure 10:**
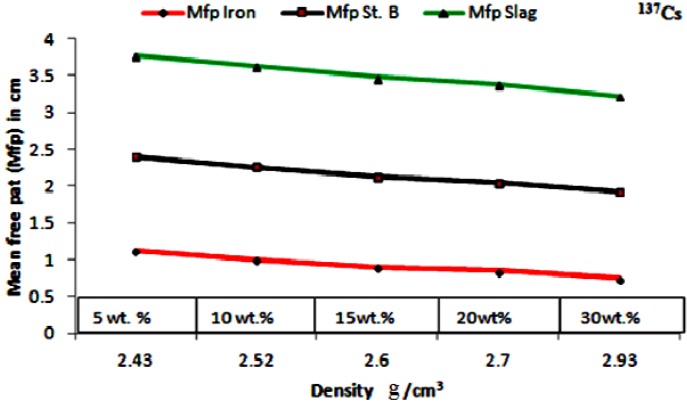
The measured mfps for concrete samples at different loading rates of iron fillings, steel balls and slags for the ^137^Cs source against the bulk density of the samples.

The plots in Figure 10 are of mfp against the bulk density for different concrete samples at different loading rates of iron fillings, steel balls and slags. The steel slags samples showed the highest mfp values. These attributed to the low specific gravity of the steel slags (2.2 to 2.5). The good dispersion of the steel slag powder within the concrete samples failed to compensate for its inferior density in attenuation performance.

## 4. Conclusions

During this study, strict screening, sizing, cleaning and classification procedure for slags and other different iron particulates addition were implemented.

(1) The maximum linear attenuation coefficients (µ) were attained for concrete incorporates iron filling wastes of 30 wt %. They were of (1.12 ± 1.31× 10^−3^) for ^137^Cs and (0.92 ± 1.57 × 10^−3^) for ^60^Co.
(2) The minimum mfp values of the examined samples were of 0.89, 1.76 and 1.32 cm for ^137^Cs source and 1.1, 1.25 and 1.4 cm for ^60^Co for iron fillings, steel balls and steel slags additives respectively at the maximum utilized loading rate of 30 wt %.
(3) The results proved that dispersion and grain size of the added particulates have great impact on attenuation performance of the concrete–additives composite samples besides the bulk density and porosity level of the solidified concrete mix.
(4) Substantial improvement of 20%–25% in attenuation performance was attained for samples of iron waste fillings as opposed to that of steel ball samples for different (5%–30%) loading rates.
(5) Iron waste fillings can be utilized successfully as an additive to anti radiation concrete to improve the structure and the bulk density of concrete and in turn its attenuation performance.
(6) Samples contained steel slags powder showed inferior attenuation performance as compared to the other samples. This finding indicates that steel slags may be useful for replace Portland cement as cementitious material rather than as density promoter.
